# On the Use of Creosote in Mercurial Salivation

**Published:** 1848-11

**Authors:** E. W. Faulcon

**Affiliations:** Warren county, N. C.


### *On the Use of Creosote in Mercurial Salivation.* By E. W. Faulcon, M. D., of Warren county, N. C.

To the Editor of the Medical Examiner.

Dear Sir,—Two days ago, while looking over the last edition of "Watson's Practice of Physic," on the subject of "Mercury in Inflammation," I recalled to memory a case that came under my observation six years ago, while I resided in another section of this State. It was one of severe salivation from the use of this mineral in remittent bilious fever. Dr. Watson says, "I have tried all sorts of expedients, and I have asked a great number of my friends what is the best plan to adopt in such cases, but I never could get satisfactory information from them. Some thought purging was the best thing: others recommended alum gargles, and gargles made of the chloride of soda: some recommended sulphur and a few iodine: all admitted that they knew of no certain remedy: neither do I."

On the 25th September, 1842, I was called in consultation to J. S., who was suffering from severe salivation, and as *some* of the usual remedies in such cases had been used without benefit in this disease, (for such it certainly is,) and as the muscles of the jaws were rapidly stiffening, so as scarcely to permit his speaking with distinctness, I suggested to his attending physician the use of the following gargle: creosote ʒss., sage tea one pint. To be used every hour during that day, and its effects accurately noted. In thirty minutes after its first application he felt a sharp tingling sensation along the angles of both jaws, and a slight convulsive motion in the muscles of the lower jaw. Shortly after the appearance of this last sensation, there was a marked relaxation of all the muscles of the face, and he expressed himself as feeling better (locally) than he had done for many days. In the evening there was a very great decline of the salivary discharge, and a great improvement in the appearance of the mucous membrane of the mouth and palatine region, which, prior to its application, wore that aspect so indicative of a near approach to sloughing. On the 26th, treatment continued, but with a slight variation or increase of the amount of sage tea. 27th.—Still continues to improve, the discharge nearly arrested: 28th.—Improving—continue treatment: 29th.—Improving, discontinue creosote but add to the strength of the sage tea: 30th.—So much improved as to require no further local treatment. Directed that the bowels be kept soluble by the use of mush and milk as diet, and occasionally aperient doses of the phosphate of soda. In a few days thereafter, he was "dismissed cured," or in other words, to receive no more visits from his physician. As creosote has some celebrity as a hæmostatic in some of the "morbi externi," I reasoned from these premises, that it might prove of service in this peculiar condition of the salivary organs. True, it is but a *single* case, yet so manifest were the benefits derived from its use, that I would not fail to resort to it in similar cases, nor to commend it, as I now do, to the members of our craft, with the hope and belief that *their* experience with it, will accord with my own. Such is the hasty account of its use, which you may dispose of as your judgment may direct.

*August* 16*th*, 1848.

